# Spinal and supraspinal mechanisms of chronic itch: from neuronal circuits to the neuro-immune-microbial axis

**DOI:** 10.3389/fmed.2026.1810269

**Published:** 2026-06-02

**Authors:** Shengrun Gao, Mengyan Liu, Ziyue Qi, Yuheng Li, Yu Xue, Chengjie Gao

**Affiliations:** 1 Department of Anesthesiology, The 960th Hospital of PLA, Jinan, China; 2 School of Anesthesiology, Shandong Second Medical University, Weifang, China; 3 Medical Service Training Center, The 960th Hospital of PLA, Jinan, China

**Keywords:** chronic itching, glial cells, GRPR+ neurons, gut-spinal axis, neural circuits, pain-pruritus interaction, pruritus, spinal cord

## Abstract

Itch is an unpleasant sensation that evokes scratching behavior; when it becomes chronic, it severely impairs quality of life, and current therapies have limited efficacy against non-histaminergic itch. The spinal cord serves as a key hub for itch integration, mediating chemical and mechanical itch through distinct neuronal subpopulations such as gastrin-releasing peptide (GRP) and its receptor (GRPR^+^), neuropeptide Y1 receptor (NPY1R^+^), and urocortin 3 (UCN^3+^) neurons, and is also regulated by inhibitory microcircuits and glial cells. Recent studies have shown that remodeling of spinal itch circuits depends not only on local neurons and glial cells but also on remote regulation by systemic signals, including peripheral immunity and the “gut-spinal cord axis,” forming a multi-level neuro-immune-microbial network. Meanwhile, chronic inflammation can amplify or convert itch signals even before they enter the spinal cord by reshaping peripheral-spinal pathways, altering immune cell functions, and modulating epithelial-neural interactions. At supraspinal levels, the ventral tegmental area (VTA)–nucleus accumbens (NAc) reward circuit encodes the pleasure and motivation associated with scratching, driving the “itch-scratch” cycle. Descending regulatory pathways constitute an important anti-itch system: Tac1^+^ glutamatergic neurons in the periaqueductal gray promote scratching, whereas the cortex (rostral anterior cingulate cortex [rACC]) activates periaqueductal gray GABAergic neurons through excitatory projections; these, in turn, descend via the rostral ventromedial medulla to the spinal cord, directly or indirectly inhibiting GRPR^+^ itch-transmitting neurons. Multiple neuronal subpopulations in the rostral ventromedial medulla, including neurokinin 1 receptor (NK1R^+^), *κ*-opioid receptor^+^ (KOR), and GPER^+^ neurons, participate in itch suppression. In addition, A11 dopaminergic neurons project descending fibers to the spinal cord and facilitate itch transmission via the DRD1^+^-GRP-GRPR microcircuit, together with the midbrain reward circuit constituting a dual dopaminergic modulation of itch. These findings extend itch regulation from local spinal circuits to a whole-brain-spinal-body network encompassing peripheral immunity, ascending reward, and descending inhibition. In-depth elucidation of itch-pain interactions, the functions of opioid receptor subtypes, and the specific pathways underlying mechanical itch is expected to facilitate translational application of targeted therapies, providing precise and effective intervention strategies for refractory chronic itch.

## 1 Introduction

Itch is defined as an unpleasant sensation that evokes a scratching impulse, exerting multifaceted effects on emotional states and motor functions (1). It can be elicited by external mechanical or chemical stimuli and is also associated with various pathological conditions, including pruritic skin disorders, cholestasis, and chronic renal failure. Like pain, itch serves as a protective response that drives organisms to detect and avoid noxious stimuli. Acute itch sensations can trigger scratching behavior, which not only alleviates the aversive stimulus but may also produce a sense of relief or pleasure. However, in chronic disease states, persistent scratching often leads to skin barrier disruption, tissue damage, and even secondary hyperalgesia due to ensuing inflammation and neural sensitization (2). While transient itch induced by brief chemical or mechanical stimuli can typically be relieved by scratching or topical agents, effective treatments for chronic, recurrent pruritus remain lacking, representing a major unmet clinical need. A deeper understanding of the neurobiological mechanisms underlying itch generation and transmission is therefore essential for elucidating its fundamental nature and guiding therapeutic development. In recent years, significant advances have been made in basic itch research, yielding breakthrough discoveries at both peripheral and central levels. The spinal cord, as a critical relay and integration center for somatosensory processing, has emerged as a key site where both neurons and glial cells actively modulate pruriceptive signaling. Building upon these recent breakthroughs, this review provides a systematic synthesis of current knowledge on spinal mechanisms of itch modulation, highlights unresolved questions, and aims to offer insights for future research and the development of novel interventions for itch-related disorders.

## 2 Peripheral mechanisms related to itch sensation

Itch can be classified into chemical and mechanical itch based on the different inducing stimuli, and chemical itch can be further divided into histamine-dependent and non-histamine-dependent subclasses according to the differential responsiveness to antihistamine drugs. Chemical itch can be evoked either by exogenous pruritogens such as chloroquine, which directly activate sensory neurons, or by pruritic stimuli that induce epithelial cells and immune cells to synthesize endogenous pruritogenic substances, thereby indirectly mediating sensation generation, while mechanical itch involves the formation and transmission of sensory information induced by non-noxious contact, electrical stimulation, and the like. The peripheral transmission of various types of itch information depends on multiple receptors distributed in the superficial layers of the skin. Most of these receptors are free nerve endings formed by unmyelinated C-type nerve fibers in the superficial skin, and some Aβ fibers are also involved in the peripheral transmission of itch. The neuronal cell bodies of such itch receptors are usually located in the dorsal root ganglia or trigeminal ganglia. They mediate signal transduction regulation through the expression of receptors such as histamine, protease-activated receptor (PAR), G protein-coupled receptor (MRGPR), toll-like receptor (TLR), 5-hydroxytryptamine (5-HT), and thymic stromal lymphopoietin (TSLP). With the development of single-cell sequencing technology, an increasing number of potential pruritus-associated receptors have been discovered, such as the interleukin (IL-31) receptor, CysLTR2, and NPBWR1, further expanding our understanding of peripheral itch mechanisms. Histamine is the earliest substance identified to be involved in itch regulation. It is usually produced by mast cell degranulation and can act on four widely distributed receptors, HRH1–HRH4, promoting itch sensation by directly activating histamine receptors expressed on neurons (3, 4). PAR receptors use exogenous or endogenous proteases as ligands and are mainly distributed in epithelial cells, immune cells, and neurons. Their activation requires two steps: hydrolysis of the N-terminal tethered ligand of the protease molecule and autoactivation of the hydrolyzed product, potentially exerting indirect regulatory effects on itch by activating MRGPR-class receptors (3). MRGPR-class G protein-coupled receptors are highly conserved in evolution, can detect stimuli from both endogenous and exogenous pruritogens, and are closely related to the regulation of histamine-dependent chemical itch. Knockout of these receptors using genetic engineering techniques leads to reduced sensitivity to pruritogens in experimental animals (3, 5). TLRs are widely distributed in skin cells, immune cells, and nerve endings. They not only mediate itch sensation induced by pruritogens, but their TLR3 and TLR7 subtypes can also participate in the chronic persistence of itch by regulating the excitability of pruriceptive neurons (3, 5). 5-HT can be released by mast cells. In skin tissue, its itch-related receptors are expressed in the peripheral terminals of dorsal root ganglion (DRG) neurons and in immune cells. Knockout of the Htr7 gene, leading to receptor dysfunction, can inhibit itch sensation (4, 6). TSLP binds to the IL-7 receptor IL-7α via its receptor in the DRG to form the IL-7α/TSLPR complex, participating in the regulation of pruriceptive neuron function. Intradermal injection of TSLP or activation of TSLPR on DRG can both lead to the generation of itch sensation. The above-mentioned receptors are distributed in three subpopulations (NP1–NP3) of non-peptidergic neurons, in C-fibers that express TSLPR, and in Aβ-type low-threshold mechanoreceptor nerve fibers that express TLR5. Under the influence of endogenous or exogenous pruritogenic factors, the levels of substances such as cathepsin S, substance P, sphingosylphosphorylcholine, microRNA miR-711, interleukin (IL)-4, and IL-31 increase. These substances further regulate the excitability of nerve cells by modulating the permeability of ion channels expressed in nerve fibers, such as Nav1.9, TRPV1, TRPA1, and Piezo2 (7, 8), thereby activating the signaling pathways of the aforementioned receptors and facilitating the transmission of itch information to the spinal cord.

Recent studies have revealed that chronic inflammation can remodel the peripheral-spinal itch pathway, altering the spinal cord’s response to specific pruritic signals. In an atopic dermatitis model, sensory neurons upregulate IL-31 receptor A (IL-31RA) expression via signal transducer and activator of transcription 3 (STAT3) signaling, thereby enhancing sensitivity to IL-31 and driving persistent scratching behavior; specific knockout of STAT3 in sensory neurons significantly reduces IL-31RA expression and markedly alleviates scratching behavior (9). Meanwhile, under the influence of chronic skin inflammation, the traditional mast cell–histamine pathway becomes less prominent, and basophils become the main effector cells in acute itch episodes. Upon allergen challenge, basophils release leukotriene C4 (LTC4), which acts on the highly expressed CysLTR2 receptor in spinal projection neurons and activates downstream signaling via TRPV1/TRPA1 channels, triggering vigorous scratching, a process in which mast cells are not involved (10). Non-neurogenic pruritogens can also indirectly affect spinal itch processing through epithelial–neural interactions. In a psoriasis model, keratinocytes highly express the TRPV4 channel, which regulates ATP release and neuropeptide secretion, thereby activating and amplifying the IL-23/Th17 immune response and driving chronic inflammation and associated itching (11). Notably, a recent study integrating single-cell electrophysiology and transcriptomics (Patch-seq) with spatial transcriptomics has identified a C-OSMR-SST neuronal subpopulation in humans and pigs. This subpopulation highly expresses OSMR, SST, and SCN11A (Nav1.9), and its electrophysiological characteristics—such as pronounced activity-dependent slowing, sine wave preference, and low high-frequency following capability—closely match those of classical mechanically insensitive C-fibers (CMi-fibers), that is, “sleeping nociceptors.” Furthermore, it was demonstrated in humans that its ligand, oncostatin M (OSM), selectively modulates the conduction properties of CMi-fibers and enhances histamine-induced flare responses without affecting mechanosensitive C-fibers (12). Together, these findings indicate that spinal integration of chemical itch relies not only on local circuits but also on remote regulation by peripheral immune status and aberrant epithelial cell activation. Moreover, they suggest that in the human itch system, specific molecularly defined sensory neuron subpopulations, such as OSMR^+^/SST^+^ neurons, constitute the structural basis for peripheral signal input, and that the “chemical itch” signals received by the spinal cord may originate from a multicellular interaction network following skin barrier disruption, rather than solely from degranulation of a single immune cell type.

## 3 Spinal neuronal mechanisms regulating chemical itch

**Figure 1:**
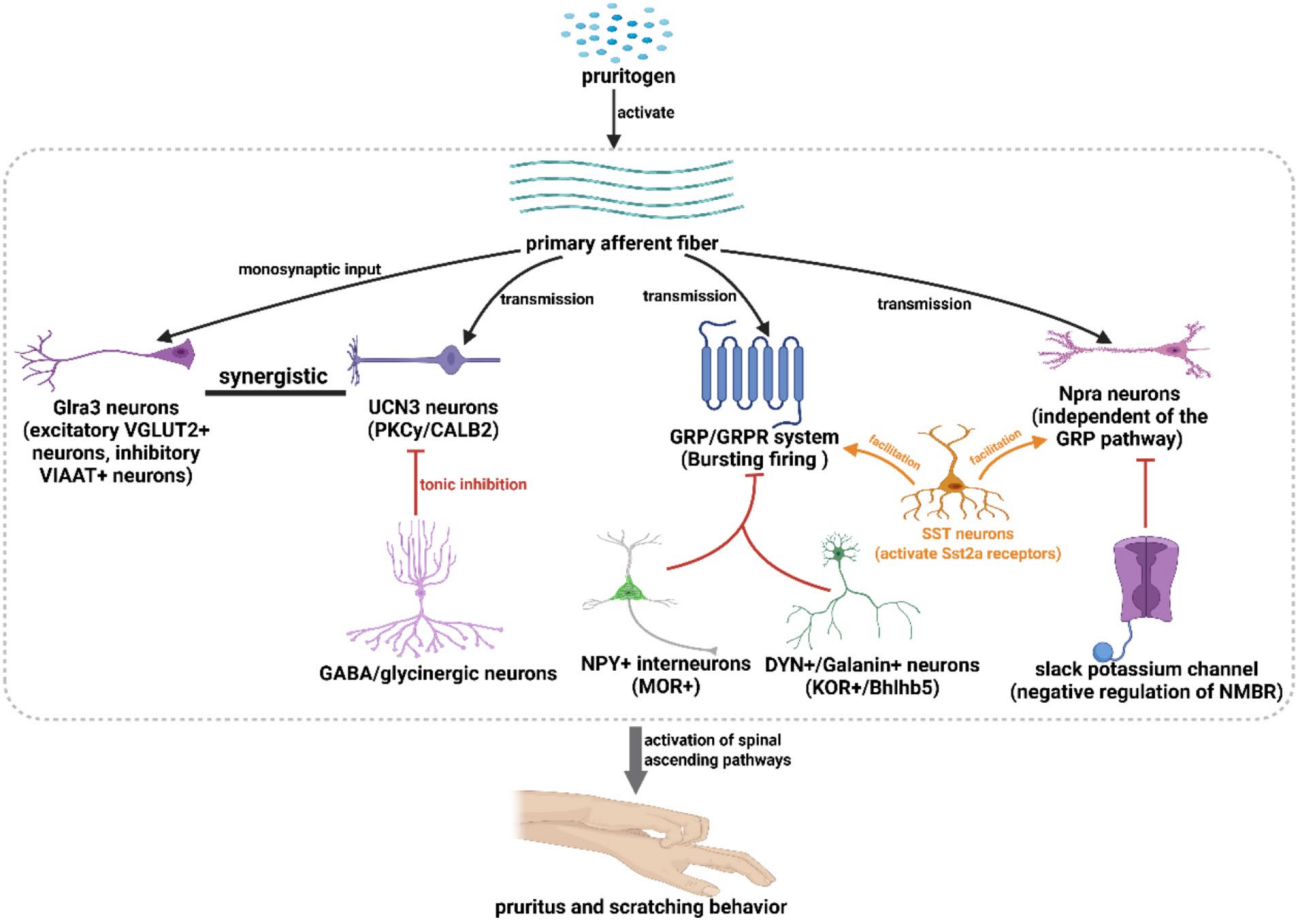
Regulatory mechanisms of spinal neurons in chemical itch. Created in BioRender (https://BioRender.com/kywb3pw).

At the spinal level, the mechanisms and regulatory processes underlying chemical and mechanical itch differ significantly. This section first summarizes the regulatory mechanisms of chemical itch. The regulation of chemical itch in the spinal cord includes facilitatory modulation of excitatory transmission of peripheral signals and inhibitory regulation mediated by certain interneurons (Figure 1).

Multiple excitatory neurons play important roles in the modulation of chemical itch information in the spinal cord. Knocking out key genes such as Tlx3 causes neuronal functional deficits, leading to impaired function of multiple types of excitatory neurons in the superficial dorsal horn of the spinal cord, resulting in significantly reduced scratching behavior induced by pruritogens (13). With continuous deepening of research, the regulatory mechanisms of various specific excitatory neurons involved in chemical itch transmission have been successively revealed. Spinal neurons expressing gastrin-releasing peptide (GRP) and its receptor GRPR are currently one of the most thoroughly studied classes of excitatory neurons that mediate the transmission of chemical itch information. The location of GRP and its receptor GRPR within the nervous system was once controversial. Earlier studies have indicated that GRP existed only in spinal dorsal root ganglion neurons; after dorsal root ganglion ablation in experimental animals, GRP staining in DRG was significantly reduced, and itch-related behaviors caused by upregulation of its expression due to various stimuli could also be suppressed by the surgery (14). However, subsequent studies have successively confirmed through qPCR, *in situ* hybridization, and genetic editing in experimental animals that GRP/GRPR is sufficiently expressed in superficial dorsal horn neurons of the spinal cord (15, 16) and plays an important role in itch regulation. A study published in Nature has demonstrated that after knocking out spinal GRPR^+^ neurons in mice, scratching behavior induced by pruritogens could be almost completely inhibited (17); pharmacological activation of GRPR could induce scratching behavior, and downregulating GRP expression levels in the spinal cord of experimental animals via transgenic techniques could significantly reduce scratching and itch-relief behaviors induced by chemical stimuli (18). This experimental evidence directly confirms the important role of the GRP system in spinal transmission of chemical itch information.

With the advancement of neuroscience research technologies, cutting-edge methods such as optogenetics have also been applied to itch research. Researchers can use this technology to achieve real-time and precise regulation of these neurons. Some researchers have found that using optogenetics to activate spinal GRPR^+^ neurons could directly induce scratching and licking behaviors in mice caused by itch, further confirming the role of these neurons in spinal itch regulation (19). Based on multiple studies clearly demonstrating that spinal GRP/GRPR neurons participate in chemical itch regulation, Pagani et al. (20) used patch clamp techniques to further investigate the electrophysiological properties of these neurons in chemical itch regulation. Their results indicated that excitation of presynaptic neurons inducing the formation of a single action potential is insufficient to excite postsynaptic GRPR^+^ neurons and generate action potentials, thereby blocking the upward conduction of itch information; only when presynaptic neurons fire in burst mode can GRPR^+^ neuron depolarization be induced, triggering itch-related behavioral changes in animals (20). This body of evidence collectively establishes the critical role of the GRP/GRPR system in chemical itch transmission.

Additional studies have demonstrated that this regulatory process critically depends on two NPY-related inhibitory microcircuits: on the one hand, NPY^+^ inhibitory interneurons in the spinal dorsal horn (SDH) express mu-opioid receptors (MOR); activation of MOR by opioids inhibits their excitability, thereby disinhibiting downstream GRP^+^ excitatory neurons. GRP^+^ neurons highly co-express NPY1R; upon activation, they release GRP, which acts on GRPR^+^ neurons to transmit itch signals. The NPY ^-^NPY1R system can directly inhibit the excitability of GRP^+^ neurons, forming negative regulation (15); on the other hand, NPY^+^ neurons form direct synaptic connections with GRPR^+^ neurons, releasing NPY to act on Y1 receptors on the surface of GRPR^+^ neurons, enhancing inhibitory postsynaptic currents (IPSCs) to suppress their activity. In chronic itch states, Y1 receptor expression on GRPR^+^ neurons is significantly downregulated, and both the frequency and amplitude of IPSCs are synchronously reduced, leading to weakened NPY-mediated inhibition, overactivation of GRPR^+^ neurons, increased sensitivity of the organism to chemical pruritogens such as chloroquine, and ultimately induced chemical itch hypersensitivity (21).

The spinal regulation of chemical itch is a complex process that involves the coordinated action of multiple neuronal subpopulations, key molecular channels, and specific ascending circuits. Different functional modules, through signal integration and precise transmission, jointly constitute the itch regulatory network from the spinal cord to higher centers. In addition to the core regulatory pathways, multiple functionally complementary neuronal subpopulations also participate in regulation. Excitatory neurons expressing urocortin 3 (UCN3) in the lumbar spinal cord serve as a hub for chemical itch transmission at the spinal level. These neurons are divided into two non-overlapping subpopulations expressing PKCγ and CALB2, respectively. They receive monosynaptic input from C-fibers carrying chemical pruritic signals and are under tonic inhibition by local glycinergic and GABAergic neurons, achieving dual regulation of itch signals. The chemical pruritogen compound 48/80 can significantly activate this neuronal subpopulation (as evidenced by elevated fos gene expression), and chemogenetic inhibition of UCN3 neurons markedly reduces bite-and-lick itch-related behaviors induced by compound 48/80; conversely, activation of these neurons directly elicits targeted itch behaviors. Thus, UCN3 neurons, by integrating peripheral chemical pruritic signals and local inhibitory control, lay the foundation for initial processing of itch signals within the spinal cord (22). Cooperating with UCN3 neurons are neurons expressing the glycine receptor α3 subunit (Glra3). These neurons are mainly distributed in laminae III–VI of the spinal dorsal horn and also include both excitatory (Vglut^2+^) and inhibitory (Viaat^+^) subpopulations. They receive monosynaptic input from peripheral itch afferent fibers and respond to glycine signals. Chemical pruritogens such as compound 48/80 and chloroquine can activate spinal Glra^3+^ neurons and upregulate their fos gene expression, subsequently triggering scratching behavior. Chemogenetic inhibition of these neurons significantly attenuates itch responses induced by the aforementioned pruritogens. Furthermore, Glra^3+^ neurons form synaptic connections with primary itch-related afferent fibers expressing Mrgpra3, SST, natriuretic polypeptide b (Nppb), etc., specifically mediating chemical itch transmission from hairy skin, and together with UCN3 neurons, they constitute the key cellular basis for chemical itch signal processing within the spinal cord.

Some studies have found that neurons expressing the receptor for natriuretic polypeptide b (Nppb), Npra, play an important role in the regulation of chemical itch. Researchers inhibited spinal dorsal horn Npra^+^ neurons and observed inhibitory regulation of chemical itch induced by pruritogens. At the same time, inhibition of these neurons did not affect itch-related behaviors induced by intrathecal injection of GRP, suggesting that the functions of these two cell types in regulating chemical itch are mutually independent (15). Somatostatin (SST)-expressing neurons are distributed in both primary afferent neurons and the spinal dorsal horn. Using gene-editing methods to cause experimental animals to lack SST leads to reduced responsiveness to chemical itch stimuli; conversely, activation of Sst2a receptors in the spinal dorsal horn enhances GRP- or Nppb-mediated itch behaviors (23), indicating that SST exerts a facilitatory regulatory role in chemical itch modulation. At the molecular regulatory level, Slack potassium channels, which are highly expressed in spinal dorsal horn neurons, play an important negative constraining role. These channels are mainly distributed in laminae I^−^III, where they co-express with the excitatory neuron marker VGLUT2 in 80.8% of cases. Additionally 96.4% of neuromedin B receptor (NMBR)-positive neurons also express Slack. Slack suppresses excessive itch responses by limiting neuronal excitability. Specific knockout of Slack in the spinal dorsal horn (Lbx1-Slack^−^/^−^ mice) significantly enhanced scratching behaviors induced by multiple chemical pruritogens such as histamine and chloroquine. Additionally, it markedly exacerbated itch responses triggered by intrathecal injection of neuromedin B (NMB). However, it had no effect on GRP-induced itch behaviors, indicating that it can selectively regulate chemical itch signal transmission mediated by the NMB/NMBR pathway and prevent over-amplification of itch responses (24).

The spinal cord also contains a large number of inhibitory interneurons that exert negative regulatory effects on the transmission of chemical itch information. When their function becomes abnormal, the neural pathways mediating itch transmission enter a state of disinhibition, which can induce itch-related behaviors such as scratching. Knockout of the atonal-related transcription factor Bhlhb5 significantly reduces the number of neurons expressing galanin and neuronal nitric oxide synthase (nNOS) in the spinal cord, leading to a marked increase in mouse responses to chemical pruritogens, suggesting that spinal galanin^+^ and nNOS^+^ inhibitory interneurons exert negative regulation on chemical itch (25). A recently published study further indicated that there is an inhibitory synaptic connection between galanin^+^ neurons and spinal GRPR^+^ neurons, further revealing the neural basis for their inhibition of chemical itch transmission (26). However, the mechanism by which nNOS^+^ neurons mediate inhibitory regulation of chemical itch still requires further investigation. Interneurons expressing dynorphin (DYN) also express Bhlhb5. Some researchers have investigated the role of DYN^+^ neurons in itch regulation, and their results showed that noxious stimuli promote VGlut^2+^ primary sensory neurons to release the excitatory neurotransmitter glutamate, thereby activating spinal DYN^+^ neurons, which then exert inhibitory regulation on GRPR^+^ neurons that facilitate chemical itch, thus producing an anti-pruritic effect. Simultaneously, knockout of the Vglut2 gene in peripheral nociceptors expressing Nav1.8 eliminates the facilitatory effect of peripheral stimulation on DYN^+^ neurons, resulting in suppressed pain perception but enhanced chemical itch sensation. These studies not only reveal the inhibitory regulatory mechanisms of chemical itch but also provide a preliminary explanation for the phenomenon of “pain inhibiting itch” (9). Building on these findings, other researchers used pharmacological tools to inhibit the opioid kappa receptor (KOR), the receptor corresponding to DYN, and found that this treatment increased scratching behavior in experimental animals; however, when Npra^+^ excitatory neurons were knocked out, this effect was attenuated, suggesting that activation of DYN^+^ neurons may inhibit chemical itch by suppressing Npra^+^ neurons (27). Interestingly, the mu-opioid receptor (MOR) plays an opposite role in itch regulation. A study published in 2021 has confirmed that activation of MOR on GABAergic inhibitory interneurons places GRPR^+^ neurons in a disinhibited state, leading to enhanced itch sensation (28). The difference in expression locations of MOR and KOR may be the reason for their divergent roles in itch regulation. These experimental results suggest that opioid receptors expressed on different types of spinal neurons exhibit differential effects in itch modulation, which may provide implications for the clinical application of such drugs. GABAergic neurons are an important class of inhibitory nerve cells. Some researchers have found that using pharmacological tools to activate spinal GABAA interneurons could significantly alleviate itch-related behaviors (29). In addition to the above regulatory mechanisms, spinal glycinergic neurons also exert inhibitory regulation on itch; using pharmacological tools to activate these interneurons can significantly suppress chemical itch sensation (30).

Notably, the majority of the mechanisms described in this section are derived from mouse studies. However, recent cross-species comparisons have revealed certain differences between the peripheral itch-sensing systems of humans and mice. With respect to MRGPR family receptors, humans lack the MrgprA/C subfamilies that are abundant in mice but possess the primate-specific MRGPRX subfamily. The MRGPRD gene is conserved in both species, but its co-expression patterns differ—in human DRG, MRGPRD and MRGPRX1 are co-expressed in approximately one-third of TRPV1^+^ neurons (MRGPRX1 expression proportion 32.5% and MRGPRD expression proportion 36.5%) (31). Regarding expression of itch-associated neuropeptides, RNA-Seq studies have shown that Nppb is highly expressed in mouse DRG (24.1 RPKM), whereas its expression levels are significantly lower in human and rat DRG (both <1 RPKM), suggesting that the role of Nppb as a primary afferent itch transmitter may exhibit species differences (32). Furthermore, Sheahan et al. (33) have confirmed the expression of NK1R (TACR1) mRNA in the human spinal cord using *in situ* hybridization and have found that TACR1 is predominantly located in the superficial dorsal horn (SDH) in humans, a distribution pattern significantly different from that in mice. In addition, using a mouse model, the same study has further demonstrated that NK1R and GRPR are co-expressed in excitatory interneurons of the superficial dorsal horn and that these neurons participate in itch regulation. These findings provide important evidence for understanding the molecular basis of spinal itch circuits and suggest that molecular targets for itch regulation may exhibit species specificity. Therefore, caution is warranted when extrapolating results from animal studies to clinical applications.

Pruritogens activate primary afferent fibers, which act on Glra3 neurons, UCN3 neurons, the GRP/GRPR system, and Npra neurons via monosynaptic input and signal transmission, respectively. GABAergic/glycinergic neurons exert tonic inhibition on UCN3 neurons. NPY + interneurons and DYN+/galanin+ neurons inhibit the GRP/GRPR system, while SST neurons (activating Sst2a receptors) facilitate signal transmission to the GRP/GRPR system and Npra neurons. Additionally, Slack potassium channels negatively regulate NMBR. Collectively, these signals activate spinal ascending pathways, ultimately eliciting pruritus and scratching behavior.

## 4 Spinal neuronal mechanisms regulating mechanical itch

**Figure 2:**
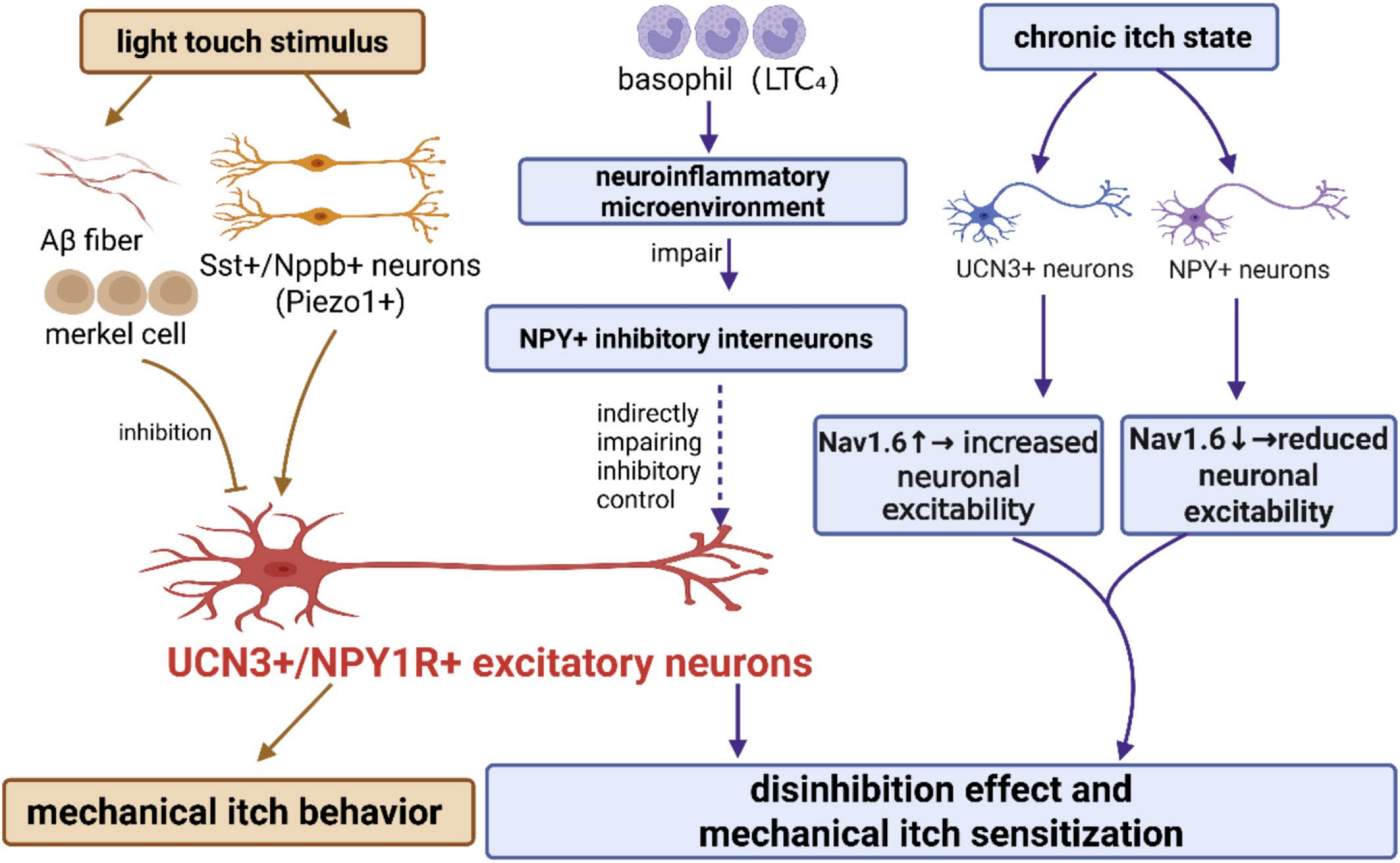
Regulatory mechanism of spinal neurons in mechanical itch. Created in BioRender (https://BioRender.com/jfkmpxb).

In addition to chemical itch induced by endogenous or exogenous pruritogens, light tactile stimuli are also transmitted to the central nervous system via cutaneous Merkel cells and Aβ nerve fibers. Under normal physiological conditions, Merkel cells and their Piezo2 channels inhibit the conversion of light touch into mechanical itch by mediating sustained firing of SAI afferent fibers; when the function of these cells or channels is lost, light touch evokes itch (34). Recent studies have revealed that the mechanically activated ion channel PIEZO1 is selectively and highly expressed in Sst^+^/Nppb^+^ itch sensory neurons and serves as the primary mediator of mechanically activated currents in these neurons. Knockout of PIEZO1 in sensory neurons significantly reduces mechanical itch and alloknesis, whereas expression of a gain-of-function PIEZO1 mutant or direct administration of the agonist Yoda1 can enhance or induce acute itch (35). Its sensory conduction does not depend on key cells in the chemical itch transmission pathway, such as GRPR^+^ spinal neurons, showing significant differences from chemical itch regulation. At the spinal level, there are also excitatory and inhibitory regulatory neurons that mediate facilitation and inhibition of mechanical itch, respectively (Figure 2).

Spinal excitatory neurons that express neuropeptide Y1 receptor (NPY1R) are crucial in mediating spinal regulation of mechanical itch. Projection from cutaneous low-threshold tactile receptors onto these neurons can produce pruritic effects. Direct activation of spinal NPY1R^+^ neurons can induce immediate scratching behavior in animals in response to tactile stimuli, while pharmacological inhibition of their function significantly reduces scratching behavior evoked by tactile stimulation. Similarly, using genetic engineering techniques to reduce the number of these neurons achieves the same anti-pruritic regulatory effect (36). Urocortin 3 (UCN3) also plays an important role in facilitating conduction of mechanical itch. UCN^3+^ excitatory interneurons receive projections from TLR5^+^ Aβ nerve fibers and mediate the transmission of itch information to higher centers. Pharmacological or gene-editing approaches to inhibit their function or reduce their numbers can significantly weaken itch behaviors induced by light tactile stimulation. Notably, some studies have shown that UCN3 and NPY1R are co-expressed in certain spinal neurons, suggesting that they may jointly participate in spinal regulation of mechanical itch, although their specific differences still require further investigation (37).

Spinal neuropeptide Y^+^ (NPY^+^) neurons exert a regulatory effect on mechanical itch that is completely opposite to that of NPY1R^+^ neurons. Either using antagonists to block the activation state of NPY^+^ neurons or artificially reducing the number of spinal NPY^+^ neurons can lead to increased scratching behavior in response to tactile stimuli, and this phenomenon cannot be reversed by inhibiting the function of GRPR^+^ neurons (38), suggesting that the inhibitory regulation of mechanical itch by spinal NPY^+^ neurons is independent of their inhibitory role in chemical itch. Furthermore, inhibitory synaptic connections between NPY^+^ inhibitory neurons and NPY1R^+^ or UCN^3+^ neurons have been experimentally confirmed. Activation of NPY^+^ neurons suppresses the activation of NPY1R^+^ and UCN^3+^ neurons, thereby indirectly exerting inhibitory regulation on mechanical itch stimuli (36, 37). In chronic itch states, the excitability of UCN^3+^ neurons is significantly increased (upregulation of Nav1.6 and Cav2.3 channels, downregulation of SK3 channels), while the excitability of NPY^+^ neurons is decreased (downregulation of Nav1.6 and BK channels), resulting in a disinhibitory effect. Computational modeling further confirms that reduced Nav1.6 conductance in NPY^+^ neurons is a key mechanism of circuit sensitization, which significantly enhances the responsiveness of UCN^3+^ neurons to Aβ fiber input, ultimately disrupting mechanical itch homeostasis and triggering mechanical itch sensitization (39).

Further studies have revealed that systemic immune status can also remotely regulate mechanical itch sensitivity. In atopic dermatitis models, circulating basophils release leukotriene C4 (LTC4), which acts primarily on CysLTR^2+^ neurons to mediate acute chemical itch; however, the resulting neuroinflammatory microenvironment may impair the function of spinal inhibitory interneurons (such as NPY^+^ neurons), indirectly weakening the inhibition of the UCN^3+^/NPY1R^+^ pathway and thereby exacerbating mechanical itch sensitization (10). Compared with chemical itch, research on mechanical itch is relatively limited, leaving substantial room for further mechanistic exploration. These new findings emphasize that in understanding mechanical itch, it must be considered within the broader context of chronic inflammation and systemic regulation, rather than viewed in isolation as merely a spinal local circuit phenomenon.

Under light touch stimulation, signals are transmitted via Aβ fibers, Merkel cells, and Sst+/Nppb+ neurons expressing Piezo1+, wherein Aβ fibers and Merkel cells exert an inhibitory effect on UCN3+/NPY1R + excitatory neurons. In the chronic itch state, basophils release LTC4 to establish a neuroinflammatory microenvironment that impairs NPY + inhibitory interneurons, thereby indirectly weakening inhibitory control. Concurrently, the chronic itch state acts upon UCN3 + neurons and NPY + neurons: Nav1.6 expression is upregulated in UCN3 + neurons, leading to increased neuronal excitability, whereas Nav1.6 expression is downregulated in NPY + neurons, resulting in reduced neuronal excitability. Ultimately, UCN3+/NPY1R + excitatory neurons mediate mechanical itch behavior, while the aforementioned alterations in neuronal excitability collectively induce a disinhibition effect and mechanical itch sensitization.

## 5 Spinal glial cells in itch information transmission mechanisms

**Figure 3:**
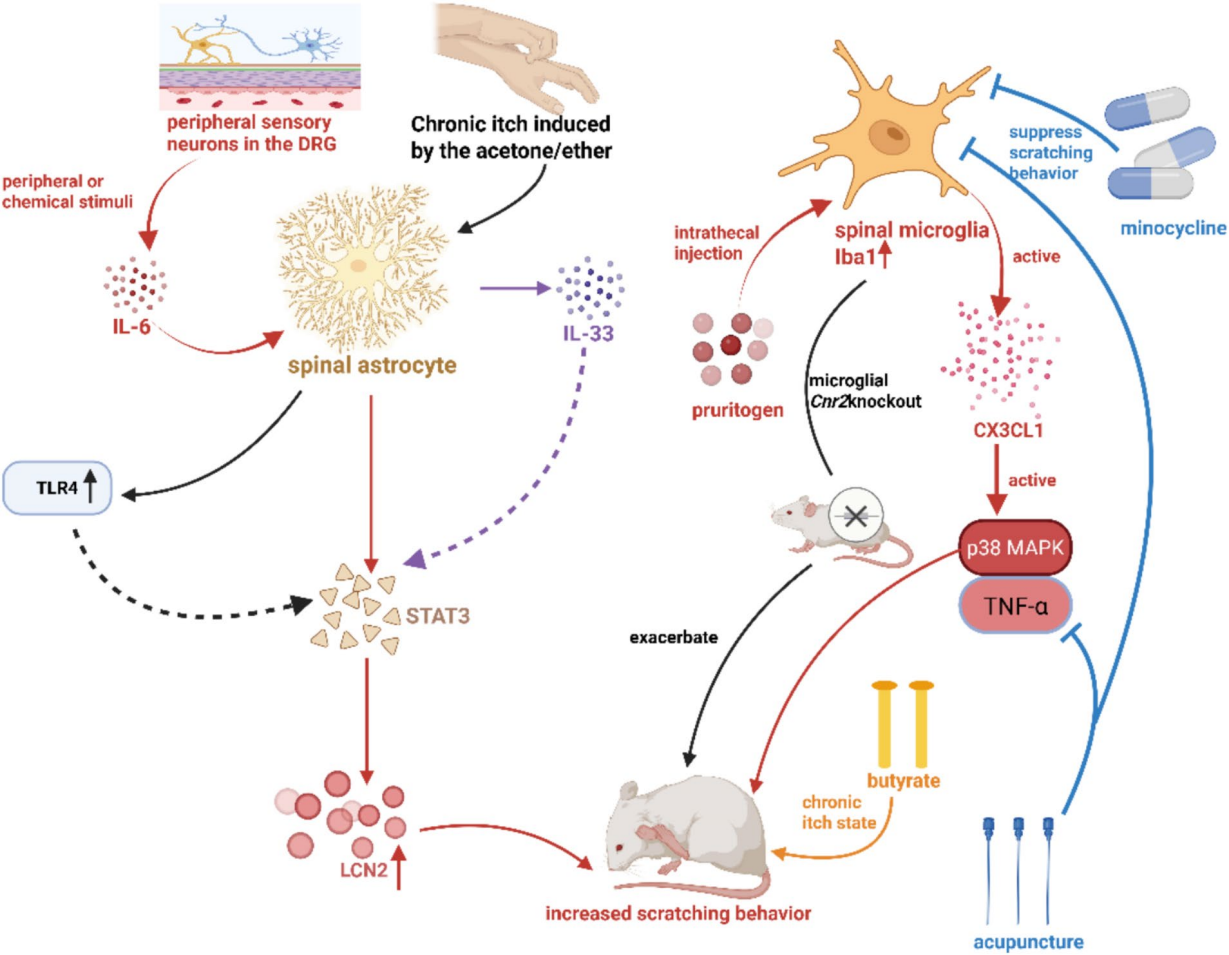
Regulatory mechanisms of spinal glial cells in itch signal transmission. Created in BioRender (https://BioRender.com/2q1zja3).

Glial cells are another major class of cells in the nervous system in addition to neurons. In mammals, their ratio to neurons can reach approximately 10:1. In the central nervous system, they are mainly classified into astrocytes, oligodendrocytes, and microglia. Glial cells present in the nervous system not only provide physical support but also participate in various neural physiological and pathological activities—including neurotrophic support, immunity, protection, and regeneration—through substance synthesis and metabolism. To date, numerous studies have shown that spinal astrocytes and microglia also participate in itch regulation (see Figure 3).

Astrocytes are the most abundant type of glial cells. They become activated in response to nervous system injury or dysfunction and participate in regulating pathological changes in the nervous system by modulating gene expression and mediating inflammatory responses. In spinal regulation of itch, the signal transducer and activator of transcription 3 (STAT3)-mediated inflammatory signaling pathway expressed by spinal astrocytes occupies a central position. In chronic itch models such as atopic dermatitis and contact dermatitis, the expression of the astrocyte-specific marker GFAP is significantly increased in experimental animals; pharmacological inhibition of STAT3 signaling activation significantly ameliorates itch-related behaviors (40). One study has confirmed that directly downregulating the expression of lipocalin-2 (LCN2), a downstream molecule of the astrocytic STAT3 inflammatory pathway, significantly alleviates behavioral changes caused by contact dermatitis and similar diseases (41). Another study has shown that directly reducing LCN2 expression derived from astrocytes significantly attenuates uremic and morphine-induced itch-related behavioral changes by inhibiting the GRP/GRPR signaling pathway and neuroinflammation (42). IL-33 is a cytokine specifically expressed by astrocytes; intrathecal injection of IL-33 induces chemical itch and scratching behavior in experimental animals. Since deficiency of the IL-33 receptor ST2 suppresses STAT3 pathway activation, it can be inferred that the pruritogenic effect mediated by IL-33 very likely acts through activation of the STAT3 pathway (43). In addition to IL-33, the pro-inflammatory cytokine IL-6 can be released by DRG peripheral sensory neurons, mediating Ca^2+^ influx into astrocytes and further activating the astrocytic STAT3 signaling pathway, upregulating LCN2 expression, and thereby inducing itch (44). Furthermore, in an acetone/ether-induced chronic itch model, TLR4 expression in spinal astrocytes is upregulated, and knockout of the TLR4 gene suppresses itch behaviors in modeled animals (45). Since the adaptor protein MyD88 in the TLR signaling pathway can mediate STAT3 activation (46), the itch-regulatory function of TLR4 may also be achieved through the key molecule STAT3, although direct experimental evidence is still needed to confirm this hypothesis.

Microglia are immune cells residing in the central nervous system and can become activated during the acute phase of itch to mediate itch transmission. Studies have confirmed that intrathecal injection of pruritogens rapidly activates spinal microglia, leading to upregulated expression of their specific marker Iba1 (47). In a 2,4-dinitrofluorobenzene-induced itch model, microglial activation occurs on day 3 after modeling (48). In this model, spinal microglia become activated after modeling and promote itch generation by increasing secretion of the chemokine CX3CL1, which activates the mitogen-activated protein kinase p38MAPK—the same signaling pathway involved in neuropathic pain. Researchers from Taiwan induced itch behavior in experimental animals via intrathecal injection of a KOR antagonist and found that acupuncture at specific acupoints could reverse modeling-induced spinal microglial activation and tumor necrosis factor-*α* (TNF-α) release, thereby reducing animal scratching behavior (49). Since TNF-α remains a downstream molecule of the CX3CL1-p38MAPK pathway, and acupuncture exerts anti-nociceptive effects in chronic pain by inhibiting the CX3CL1-p38MAPK pathway (50), it can be speculated that the anti-pruritic effect of acupuncture may also be related to inhibition of this signaling cascade, although direct experimental evidence supporting this hypothesis is currently lacking. Xu et al. (51) used imiquimod to establish a psoriasis mouse model; after modeling, mice exhibited elevated spinal Iba1 expression and itch behaviors, while minocycline-mediated suppression of microglial activation inhibited scratching behavior. Another study has found that in mouse models of dermatitis and psoriasis, knockout of the Cnr2 gene (encoding CB2R) in microglia exacerbated itch symptoms, which critically depended on the CB₂R-SOCS3-JAK1/p38-STAT1 signaling axis (52). The above studies have indicated that spinal microglia facilitate itch regulation through pro-inflammatory signaling pathways, but relevant research remains limited and mechanisms are not yet deeply understood. Similarly, in different chronic itch models, whether mediators released by peripheral immune cells can indirectly activate spinal glial cells—either by crossing the blood–brain barrier or via neural projections—to form a “peripheral-central” positive feedback loop remains a key question urgently requiring exploration.

Astrocytes become activated in response to pruritic stimuli and promote itch behaviors via IL-6 or IL-33 signaling, which activates the STAT3 pathway and upregulates lipocalin-2 (LCN2) expression. The TLR4 pathway also contributes to this process, potentially engaging STAT3 activation through the adaptor protein MyD88. Microglia are rapidly activated during the acute phase of itch (evidenced by increased Iba1 expression), releasing CX3CL1 to activate the p38 MAPK pathway and downstream TNF-*α* signaling, thereby exacerbating itch. This microglial activation can be suppressed by minocycline or alleviated by acupuncture-based interventions. Notably, conditional knockout of the Cnr2 gene (encoding cannabinoid receptor type 2, CB2R) in microglia exacerbates itch. Collectively, astrocytes and microglia facilitate chronic itch through distinct pathways—STAT3/LCN2 and CX3CL1/p38/TNF-α, respectively—and are subject to remote regulation by peripheral immune cues and systemic metabolic signals, highlighting the intricate neuro-immune-microbial crosstalk underlying chronic pruritus.

## 6 Mechanisms of spinal itch information projection to higher brain centers

### 6.1 Neural basis of itch perception and emotional regulation

Itch stimuli detected by peripheral receptors are processed in the spinal cord and then transmitted upward to higher brain centers via distinct neural pathways. The thalamus is a key target region for spinal itch signal transmission. Electrophysiological experiments have shown that exogenous pruritogens applied to the body or head/face can activate neurons in the spinothalamic tract and trigeminothalamic tract, mediating the ascending transmission of itch information (53, 54). The parabrachial nucleus (PBN), located in the lateral reticular formation of the pontine tegmentum, is not only involved in respiration, cardiovascular, and gastrointestinal functions but has recently also been confirmed as a critical site for ascending itch transmission. Studies have demonstrated that projection neurons in the spinoparabrachial tract form direct synaptic connections with spinal GRPR^+^ neurons, suggesting their role in transmitting chemical itch; moreover, optogenetic inhibition of this projection pathway directly reduces pruritogen-induced scratching behavior (55). Additional research using *in vivo* electrophysiological techniques has confirmed that the timing of evoked activity in rat trigeminoparabrachial projection neurons in response to exogenous pruritogens closely aligns with the onset of itch-related behaviors (56), proving the important role of this pathway in ascending itch transmission from the spinal cord.

Recent studies have further revealed that the parabrachial nucleus is not merely a sensory relay station but also a key integration node for the emotional component of itch. Neurons projecting from the spinal cord to the lateral parabrachial nucleus (LPBN) transmit itch signals to the central amygdala (CeA), driving aversion- and anxiety-like emotions associated with scratching. Specific inhibition of the LPBN→CeA pathway significantly reduces anxiety behaviors induced by chronic itch without affecting the basic scratching action itself (57). Furthermore, the anterior insular cortex (AIC) has been identified as the core region encoding the subjective unpleasantness and aversive emotion of itch—selective inhibition of AIC neuronal activity significantly alleviates experimental animals’ avoidance responses and emotional distress to pruritic stimuli but does not alter their scratching frequency (58). These findings indicate that ascending itch pathways not only convey sensory intensity but also encode its affective and motivational dimensions through circuits such as LPBN-CeA-AIC, thereby explaining why chronic pruritus is frequently accompanied by significant anxiety, depression, and reduced quality of life. Another point requiring attention is that ascending projection pathways for different sensory modalities from the spinal cord exhibit overlap. Evidence has shown that most projection neurons originating from the superficial spinal cord and targeting higher centers express neurokinin 1 receptor (NK1R) as a marker; after knockout of this receptor, both itch and pain behaviors induced by pruritic and nociceptive stimuli are attenuated (59). An earlier study using non-human primates has also confirmed that the spinothalamic tract transmits multiple types of information, including itch, pain, touch, and temperature (60).

However, under chronic inflammatory conditions, the ascending itch pathway may undergo specific remodeling. For example, in atopic dermatitis models, peripheral IL-31 upregulates IL-31RA expression in sensory neurons via a STAT3-dependent mechanism, enhancing their sensitivity to pruritic signals and consequently leading to sustained activation of the spinal-parabrachial-anterior insula pathway, forming a “sensory-emotional” positive feedback loop that exacerbates scratching and anxiety (9). The functional complexity of these neural pathways may pose challenges for interventions targeting a single sensory modality. Further in-depth research into itch mechanisms may help researchers more precisely distinguish the specific functions of various neuronal populations, potentially offering positive implications for understanding disease pathogenesis and developing treatments. Particularly importantly, future targeted therapies should address both sensory conduction and emotional regulation pathways; for instance, simultaneously inhibiting the peripheral IL-31/STAT3 axis and central AIC activity might more effectively alleviate chronic pruritus and its accompanying psychological burden.

### 6.2 Regulation of itch by the dopaminergic system

**Figure 4:**
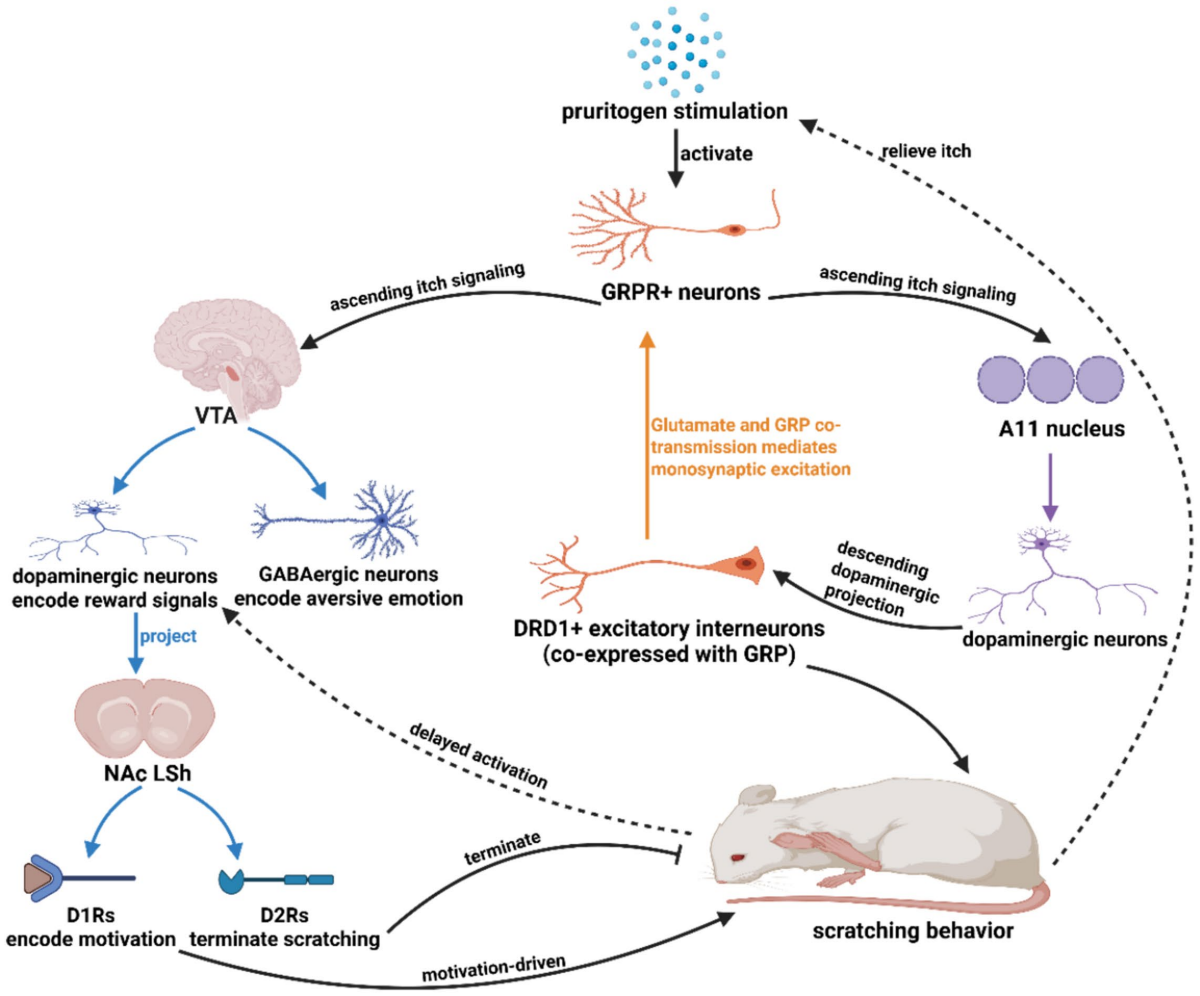
Regulation of itch by the dopaminergic system. Created in BioRender (https://BioRender.com/nbc35g4).

In recent years, studies have revealed that the dopaminergic system plays an important role in itch regulation, primarily involving the midbrain reward circuit and descending regulatory pathways (Figure 4).

Dopaminergic neurons in the ventral tegmental area (VTA) are the core of the classical reward system and play a critical role in itch processing. Su et al. (61) used fiber photometry recording to demonstrate that in acute itch models, VTA GABAergic neurons are activated immediately after the onset of scratching, encoding the aversive component of itch. In contrast, activation of VTA dopaminergic neurons is delayed by approximately 5 s and depends on the scratching behavior itself, encoding the rewarding sensation associated with scratching. Optogenetic inhibition of VTA GABAergic neurons reduces scratching behavior, whereas inhibition of VTA dopaminergic neurons disrupts the persistence of scratching, indicating that these two types of VTA neurons, respectively, regulate the aversive and rewarding components of itch, jointly maintaining the “itch-scratch” cycle.

VTA dopaminergic neurons participate in itch information processing via their projections to the nucleus accumbens (NAc). Yuan et al. (62) have found that pruritogens activate VTA dopaminergic neurons and that their projections to the lateral shell of the NAc (NAc LaSh) are strongly activated during scratching. Liang et al. (63) further used pharmacological approaches to show that blockade of D1-type dopamine receptors (D1R) in the NAc LaSh significantly reduces pruritogen-induced scratching behavior without affecting motor function, whereas activation of D2-type dopamine receptors (D2R) terminates scratching behavior. In contrast, dopamine receptors in the medial shell of the NAc (NAc MeSh) play a minor role in itch processing. Moreover, it was found that dopamine signals in D1R^+^ neurons of the NAc LaSh increase at the onset of scratching using fiber photometry combined with a dopamine sensor, suggesting that these signals encode the motivational component of scratching.

Setsu et al. (64) have confirmed that using conditioned place preference scratching behavior produces a significant reward effect in both acute and chronic itch models. Immunohistochemistry and single-cell reverse transcription polymerase chain reaction (RT-PCR) revealed that dopaminergic neurons positive for tyrosine hydroxylase (TH) and dopamine transporter (DAT) in the VTA are activated in a chronic itch model. Microanalysis experiments further demonstrated that scratching significantly increases extracellular dopamine levels in the NAc, whereas no such change occurs when scratching is restricted using an Elizabethan collar. Selective inhibition of VTA dopaminergic neurons using pharmacogenetic techniques significantly reduces the number and duration of scratching bouts, providing direct evidence for the involvement of the dopaminergic system in itch regulation.

In addition to the midbrain reward circuit, projections from A11 nucleus dopaminergic neurons to the spinal dorsal horn (SDH) constitute another important descending regulatory pathway. Zhang et al. (65) used fiber photometry recording to show that pruritogens activate A11-SDH dopaminergic neurons (dopaminergic A11-SDH), and inhibition of these neurons alleviates scratching behavior induced by acute and chronic itch. Mechanistically, D1-type dopamine receptor (DRD1)-positive neurons in the SDH are primarily excitatory interneurons and are highly co-localized with gastrin-releasing peptide (GRP) (approximately 70%). These DRD1^+^ neurons form monosynaptic excitatory connections with GRPR^+^ neurons, directly activating them through the release of glutamate and GRP, thereby facilitating the transmission of itch signals to higher brain centers. Chemogenetic activation of spinal DRD1^+^ neurons induces spontaneous scratching, whereas inhibition of these neurons reduces pruritogen-induced scratching. Furthermore, blocking AMPA glutamate receptors or GRPR abolishes scratching induced by the activation of DRD1^+^ neurons, further confirming the critical roles of GRP and glutamate in DRD1^+^ neuron-mediated itch transmission. Together, these two pathways constitute the neural circuit basis for dopaminergic regulation of itch, offering new potential targets for the treatment of chronic itch in clinical settings.

Pruritogen stimulation activates neurons that express the gastrin-releasing peptide receptor, and the ascending signals project to the ventral tegmental area (VTA) and the A11 nucleus. Within the VTA, dopaminergic neurons encode reward signals and project to the nucleus accumbens shell (NAc LSh), where they activate D1 receptors to encode the motivation for scratching. Concurrently, GABAergic neurons in the VTA encode aversive emotions. In the A11 nucleus, the signal is transmitted via dopaminergic neurons through a descending projection to excitatory interneurons expressing dopamine D1 receptors. These interneurons mediate monosynaptic excitation of neurons positive for the gastrin-releasing peptide receptor through the co-transmission of glutamate and gastrin-releasing peptide (GRP). This process forms a positive feedback loop that sustains scratching behavior. The activation of D2 receptors in the NAc LSh terminates scratching. Furthermore, the scratching behavior itself generates a reward effect by delaying the activation of dopaminergic neurons in the VTA, which ultimately relieves itch.

### 6.3 Neural circuit mechanisms of descending anti-itch pathways

**Figure 5:**
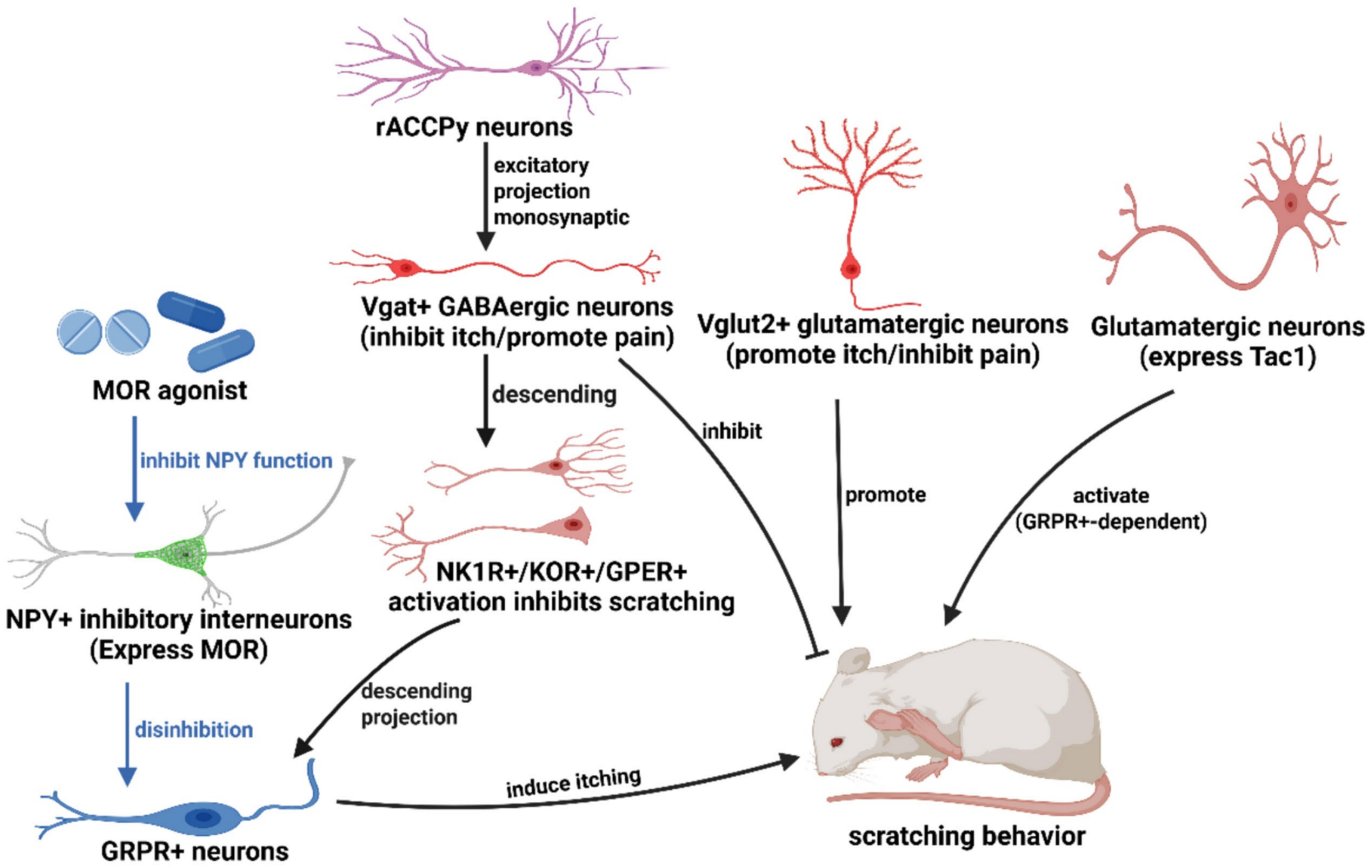
Neural circuit mechanisms of descending anti-itch. Created in BioRender (https://BioRender.com/94uodx8).

Itch signals are also subject to descending inhibitory regulation from higher brain centers, with the periaqueductal gray (PAG) and the rostral ventromedial medulla (RVM) serving as core structures (Figure 5). As the primary hub of descending regulation, the PAG was shown by Gao et al. (66) to contain Tac1-expressing glutamatergic neurons in its lateral and ventrolateral portions (I/vIPAG) that are significantly activated during itch-induced scratching behavior. Specific inhibition or ablation of these neurons reduces scratching, while their activation directly elicits scratching; this effect depends on the functional integrity of spinal GRPR^+^ neurons but does not participate in pain regulation, suggesting specificity in itch processing. Samineni et al. (67) have further revealed that glutamatergic (Vglut^2+^) and GABAergic (Vgat^+^) neurons in the PAG exert opposing regulatory effects on itch and pain—activation of glutamatergic neurons inhibits pain but promotes itch, whereas activation of GABAergic neurons promotes pain but inhibits itch.

The RVM serves as a key relay station for descending regulation from the PAG and contains multiple functionally distinct neuronal subpopulations. Traditionally, RVM neurons are classified into ON cells (pro-nociceptive) and OFF cells (anti-nociceptive) based on their responses to noxious stimuli and modulation by opioids (68). In descending inhibition of itch, several neuronal subpopulations have been implicated: activation of NK1R-expressing GABAergic neurons (corresponding to ON cells) inhibits scratching (69); GABAergic neurons expressing the *κ*-opioid receptor (KOR) constitute a descending inhibitory pathway whose activation suppresses both pain and itch (70); and GPER-expressing neurons are activated by itch stimuli, with selective ablation enhancing scratching (71). In addition to the PAG-RVM pathway, cortical-originating descending inhibitory circuits also participate in itch regulation. Wu et al. (72) have identified an excitatory descending inhibitory circuit from pyramidal neurons in the rostral anterior cingulate cortex (rACC) to PAG GABAergic neurons. rACC pyramidal neurons show reduced activity during scratching; activation of this circuit significantly suppresses scratching, while inhibition of the circuit enhances scratching. Mechanistically, rACC pyramidal neurons activate PAG GABAergic neurons via monosynaptic excitatory connections, which then inhibit itch signal transmission through the PAG-RVM-spinal cord pathway.

The spinal dorsal horn is the final effector target of descending regulation. On the one hand, Liu et al. (26) have found that GABAergic neurons in the RVM directly inhibit spinal GRPR^+^ neurons via descending projections, constituting a direct effector component of the descending anti-itch pathway. On the other hand, Wang et al. (28) have demonstrated that NPY^+^ inhibitory interneurons in the spinal dorsal horn express *μ*-opioid receptors (MOR). Activation of MOR by opioids suppresses NPY^+^ neuron function, thereby relieving the inhibition of downstream GRPR^+^ excitatory neurons (disinhibition) and consequently promoting itch. This mechanism suggests that descending regulation not only directly inhibits itch-transmitting neurons but also interacts with the local opioid system in the spinal cord to finely regulate itch signal transmission.

The descending anti-itch pathway is a multi-layered neural regulatory network. The cortex activates PAG GABAergic neurons via excitatory projections; these neurons, in turn, descend through the PAG-RVM pathway to the spinal cord, directly or indirectly inhibiting GRPR^+^ neurons. Multiple neuronal subpopulations in the RVM, including NK1R^+^, KOR^+^, and GPER^+^ neurons, participate in itch suppression through distinct transmitter and receptor mechanisms. The elucidation of these pathways provides a new theoretical basis for developing centrally targeted therapies for chronic itch.

Pyramidal neurons in the right anterior cingulate cortex (rACCPy) activate Vgat-positive GABAergic neurons in the spinal dorsal horn via monosynaptic excitatory projections. These GABAergic neurons activate neurons that express NK1R, KOR, and GPER through descending projections, thereby inhibiting GRPR-positive neurons to exert an antipruritic effect; alternatively, they directly inhibit downstream neurons to suppress scratching behavior. Notably, Vgat-positive GABAergic neurons exhibit a dual function of inhibiting itch and promoting pain. *μ*-opioid receptor (MOR) agonists act on NPY-positive inhibitory interneurons expressing MOR to inhibit their function, thereby disinhibiting GRPR-positive neurons. Meanwhile, Vglut2-positive glutamatergic neurons in the spinal dorsal horn promote scratching behavior; in contrast to Vgat-positive neurons, they facilitate itch and inhibit pain. Concurrently, glutamatergic neurons expressing Tac1 activate downstream pathways via a GRPR-positive neuron-dependent mechanism, ultimately promoting scratching behavior. Collectively, these circuits precisely regulate the initiation and inhibition of scratching behavior.

## 7 Systemic role of the neuro-immune-microbial axis in itch regulation

As research on chronic itch advances, increasing evidence indicates that itch regulation is not limited to local skin–spinal cord neural circuits but involves peripheral immunity, the central nervous system, and the gut microbiota. Studies have confirmed that the gut microbiota remotely regulates spinal microglial function via the “gut–spinal cord axis,” thereby modulating central itch sensitivity. Microglia in germ-free mice or mice treated with antibiotics exhibit an immature phenotype and show significantly reduced responses to pruritic stimuli such as chloroquine, whereas fecal microbiota transplantation restores microglial activation and itch sensitivity (73). Mechanistically, gut microbiota-derived short-chain fatty acids enter the central nervous system through the bloodstream, maintaining microglial homeostasis and promoting their transition to a pro-inflammatory phenotype under pathological conditions such as neuropathic pain, leading to the release of pro-inflammatory factors including interleukin-1β (IL-1β) and tumor necrosis factor-*α* (TNF-α) (74–76). Among these, spinal microglia activate GRPR^+^ neurons via the NLRP3 inflammasome/caspase-1/IL-1β signaling axis, with IL-1β binding to the type 1 IL-1 receptor (IL-1R1) on the surface of GRPR^+^ neurons to enhance their excitability and thereby amplify itch signals (75). Simultaneously, pro-inflammatory factors released by activated spinal microglia may also reduce the GABAergic output of inhibitory neurons, indirectly affecting various somatosensory processes, including chemical and mechanical itch (75) (Figure 3). Furthermore, activated microglia may enhance the excitability of spinal projection neurons, indirectly promoting the transmission of itch signals to the thalamus and parabrachial nucleus (73). These findings link microbial metabolism to spinal glia-mediated itch sensitization, suggesting that pruritus may not merely reflect local skin–neural circuit abnormalities but rather represents a systemic manifestation of neuro-immune-microbial network dysregulation, providing a new perspective for understanding refractory chronic itch.

## 8 Summary and outlook

Itch-related symptoms and diseases severely impair patients’ quality of life. Current therapeutic agents primarily include topical corticosteroids and local anesthetics, whose mechanisms involve blocking itch conduction at the peripheral level. Drugs targeting the central nervous system remain scarce. Further deepening of research into the central mechanisms of itch will enable researchers and clinicians to better understand its pathophysiological basis, thereby facilitating the translation of scientific findings into novel therapeutic approaches for patients suffering from chronic pruritus. Itch has become one of the hot topics in neuroscience research in recent years. With continuous accumulation of knowledge and technological advances, substantial progress has been made, leading to a more comprehensive understanding of the physiological and psychological aspects of itch. Relevant findings have been consistently published in top-tier scientific journals and have drawn significant attention from the scientific community. The spinal cord is a critical central hub for itch regulation. Numerous studies have already revealed a wealth of regulatory mechanisms underlying spinal itch processing; however, many questions remain to be explored.

Similar to the ascending projection pathways from the spinal cord to higher brain centers, certain spinal neuronal populations that mediate itch regulation also modulate pain perception. For instance, interventions targeting GRPR^+^ neurons to modulate itch also affect pain sensation (77). Glycinergic interneurons involved in inhibitory regulation of chemical itch have also been reported to exert a feedforward inhibitory “gate” function in neuropathic pain regulation (78). Clarifying whether these neurons maintain a balanced co-regulation of both unpleasant sensations—pain and itch—when confronted simultaneously, or whether there exists a temporal priority in processing these sensory signals, may further help understand and validate the hypothesis of “pain inhibiting itch.” Opioid receptors are commonly used in clinical analgesia, but their application often leads to adverse effects such as pruritus. Existing evidence has indicated that activation of MOR induces itch, whereas activation of KOR suppresses itch. Further elucidating the differences in expression locations between these two receptors may aid in novel drug development—not only enabling precise treatment for patients with pathological itch disorders but also positively influencing drug selection for patients with chronic pain, cancer pain, or postoperative pain. Moreover, well-designed basic and clinical studies could explore whether rational combination use of agonists targeting both receptor types might reduce the incidence of pruritus as a complication while effectively alleviating pain, thereby improving patients’ quality of life. It should also be noted that, compared with chemical itch, research on the mechanisms of mechanical itch remains relatively underdeveloped. Mechanical itch relies on Aβ nerve fibers, which normally transmit tactile information. Investigating whether its underlying mechanisms differ from or relate to “tactile allodynia” in neuropathic pain could represent a potential direction for future research. Current studies on the role of glial cells in itch regulation are limited, primarily focusing on their modulation of inflammatory responses. However, glial cells possess diverse functions and can mediate processes such as neural development and repair. Further clarification of the interactions between glial cells and neurons in itch regulation may help identify novel anti-pruritic targets, holding significant implications for both basic science and clinical practice.
